# Advances in anti-dengue virus properties of Traditional Chinese Medicines

**DOI:** 10.3389/fmicb.2026.1780355

**Published:** 2026-03-11

**Authors:** Ruyi Zeng, Tiantian Wu, Kui Zheng, Lu Zhang, Fangfang Sun, Shuai Yuan, Shuxiang Huang, Fan Wang, Zhiping Dai, Jian Zhou, Suxiang Li, Dandan Hu, Yongxia Shi, Jun Dai

**Affiliations:** 1 Artemisinin Research Center, Guangzhou University of Chinese Medicine, Guangzhou, Guangdong, China; 2 Guangzhou Customs District Technology Center, Guangzhou, Guangdong, China; 3 State Key Laboratory of Respiratory Disease, Guangzhou Customs District Technology Center, Guangzhou Customs District, Guangzhou, Guangdong, China

**Keywords:** dengue fever, dengue virus, research advances, *Tianxing fever*, Traditional Chinese Medicine

## Abstract

Dengue fever is an acute mosquito-borne disease caused by the dengue virus (DENV). Based on antigenic variations in the envelope (E) protein, DENV is classified into four serotypes (DENV-1 to DENV-4), with DENV-2 being most frequently associated with severe disease. Given its high transmissibility, broad geographic distribution, and rapid viral evolution, there remains an urgent need for specific antiviral therapeutics against dengue. Traditional Chinese Medicine (TCM)-characterized by multi-target pharmacology activity, generally favorable safety profiles, and a low propensity for inducing resistance—represents a promising complementary strategy for dengue prevention and treatment. Historical TCM records on *Tianxing fever*, a condition exhibiting strong clinical parallels with modern dengue, provide valuable insights for selecting candidate herbs, extraction methods, and dosage strategies. This review systematically examines herbal agents documented in classical TCM texts for the treatment of *Tianxing fever* and synthesizes contemporary pharmacological research on TCM-derived compounds with anti-dengue activity. Furthermore, it outlines key future research directions for TCM-based dengue therapeutics, with the aim of guiding clinical practice and modern drug development.

## 1 Introduction

Dengue fever (DF) is an acute infectious disease caused by the dengue virus (DENV) and transmitted primarily by *Aedes* mosquitoes. In 2019, dengue was listed among the top 10 global health threats by the World Health Organization (WHO) (Wu et al., 2022). The global burden remains substantial, with 14.1 million reported cases and 9508 deaths in 2024 alone (Haider et al., 2025). Typical clinical manifestations include acute fever, severe headache, retro-orbital pain, myalgia/arthralgia, nausea, vomiting, and rash (Frentiu, 2023); a subset of cases can progress to severe forms such as dengue hemorrhagic fever (DHF) or dengue shock syndrome (DSS) (Murphy and Whitehead, 2011). The disease imposes a significant public health and socioeconomic burden across tropical and subtropical regions, including Africa, the Americas, the Eastern Mediterranean, Soth-East Asia, and the Western Pacific (Bhatt et al., 2013; Guzman et al., 2016; Shepard et al., 2016).

To date, no specific antiviral drug has been widely approved or adopted for routine dengue treatment. Although two live-attenuated tetravalent vaccines have been licensed in certain regions, their real-world coverage and efficacy remain limited (Halstead and O’Rourke, 1977). Consequently, clinical management continues to rely primarily on supportive care, vigilant monitoring, and symptomatic treatment. Both conventional medicine and Traditional Chinese Medicine (TCM) are commonly utilized in endemic regions. Given DENV’s high transmissibility, broad geographic distribution, and rapid mutation rate, developing effective antiviral therapies remains an urgent global priority (Guzman and Martinez, 2024; Anumanthan et al., 2025). TCM, characterized by multi-target pharmacological activity, extensive historical clinical experience, and a low propensity for inducing drug resistance, represents a promising source for complementary antiviral strategies. While classical TCM texts do not explicitly mention “dengue,” modern scholars generally classify DF under the broader category of “epidemic diseases” (*yi bing*). Related historical descriptions include “epidemic rash” (*yi zhen*) and “epidemic maculopathy” (*yi ban*) (Mucheng and Shengjie, 2012). Additionally, febrile-disease literature also mentions “epidemic maculopathy” (*shu wen*) and “summer epidemic” (*shu yi*), which partially overlap with dengue‘s clinical presentation (Jiaming, 1994). Notably, historical records of “*Tianxing fever*” show striking parallel with the symptomatology of modern DF.

This review therefore aims to bridge classical TCM knowledge and contemporary science by integrating (i) historical TCM documentation related to *Tianxing fever* and (ii) modern pharmacological evidence on TCM-derived anti-dengue agents with anti-dengue activity. The synthesis is intended the objective of guiding clinical applications and future research in TCM-based dengue therapeutics.

## 2 Current Epidemiology of dengue

Dengue is the most rapidly spreading mosquito-borne viral disease worldwide, with endemic transmission sustained in over 120 countries across tropical and subtropical regions (Brady et al., 2012; Lorenzi et al., 2013; Li et al., 2017). Epidemic outbreaks typically follow cyclical patterns, peaking every 4–7 years (Wu et al., 2022).

Model-based estimates indicate that approximately 390 million dengue virus infections occur annually, of which around 96 million results in clinically apparent disease (Bhatt et al., 2013). Globally, an estimated 3.97 billion people live in areas at risk of dengue transmission (Brady et al., 2012; Diamond and Pierson, 2015). According to the WHO, 2023 recorded the highest global dengue burden on record, affecting over 80 countries and resulting in more than 6.5 million cases and 7,300 dengue-associated deaths (World Health Organization [WHO], 2024). The Pan American Health Organization (PAHO) further reported that, as of December 10, 2024, the Americas experienced a record 12.6 million dengue cases, including 21,000 severe cases and over 7,700 deaths (Wei, 2025). The global epidemic has continued to escalated in 2025. As of July 30, 2025, Brazil remains the most severely affected country, with 1.5 million confirmed cases and 1225 deaths reported as of June 18 (Yufan, 2025). In addition, significant case increases have been reported in Southeast Asia, China, Cuba, and parts of Europe. Notably, the Philippines recorded as a 40% rise in dengue incidence in February 2025 compared with the same period in 2024 (Jing, 2025).

Collectively, these data underscore that the global dengue epidemic remains a serious and escalating public health challenge, emphasizing the urgent need for enhanced surveillance, preventive measures, and the development of effective antiviral and supportive therapeutic strategies.

## 3 Clinical features

According to the Dengue Fever Diagnosis and Treatment Guidelines (2024 Edition) (National Health Commission of the People’s Republic of China, and National Administration of Traditional Chinese Medicine, 2024), the clinical course of DF can be divided into three distinct phases: the acute febrile phase, the critical phase, and the recovery phase. Although classical TCM texts do not explicitly reference “dengue fever,” they contain detailed descriptions of diseases with comparable clinical manifestations. For instance, the *Waitai Miyao* (Medical Secrets from the Royal Library), *Bencao Shugouyuan* (Essentials of Materia Medica), and *Zengding Bencao Beiyao* (Revised Compendium of Materia Medica) all document the symptoms of *Tianxing fever*, which correspond closely to those observed in modern cases of DF. Table 1 provides the symptoms of DF in classical TCM texts and modern clinical guidelines. In acute febrile phase, both dengue fever and *Tianxing fever* exhibit symptoms such as headache and congestive or petechial rash. If not treated promptly at this time, the symptoms of *Tianxing fever* will worsen, manifesting as high fever, mental confusion, and a deep, thready pulse (This indicates a deficiency of both qi and blood in TCM, which may symbolize blood loss or organ failure). This is similar to the typical manifestations of DF during its acute phase, such as high fever, hemorrhage, and shock. Although the historical records do not explicitly describe the symptoms of *Tianxing fever* in recovery, some records describe “subside muscle heat” and “resolving macular rash heat-toxicity” after patients taking the herbs to treat *Tianxing fever*. These manifestations are similar to the recovery phase of DF.

**TABLE 1:** Clinical comparison of DF and *Tianxing fever.*

Phases	Clinical manifestations	Similar description of TCM
Acute febrile phase	High fever or the classic “biphasic fever”; headache; generalized myalgia; nausea; vomiting; congestive or petechial rash.	*Bencao Shugouyuan* (Essentials of Materia Medica): “ *Tianxing fever* presents with headache, thirst, and generalized fever…” *Zengding Bencao Beiyao* (Revised Compendium of Materia Medica): “ *Tianxing fever* with delirium, and blood-heat-related pox eruptions…”
Critical phase	Occurs between days 4 and 8 of the illness, characterized by persistent high fever and, in severe cases, by complications such as hemorrhage, shock, and multi-organ impairment (e.g., liver, kidney, and heart).	*Waitai Miyao* (Medical Secrets from the Royal Library): “If *Tianxing fever* does not resolve promptly after 4–5 days, symptoms include unquenchable thirst, epigastric tension, tactile hypersensitivity, insomnia, and delirium…”; “For *Tianxing fever* persisting beyond 7 days, the fever would be intensified, accompanied by constipation, epigastric fullness, anorexia, mental confusion, incoherent speech, and a deep, thready pulse.”
Recovery phase	Fever, rash, and other symptoms gradually subside	subside muscle heat resolving macular rash heat-toxicity
Severe dengue	(1) Plasma leakage severe enough to cause dengue shock syndrome or respiratory distress. (2) severe bleeding. (3) severe organ impairment.	/

However, not all historical records describing *Tianxing fever* can be directly equated with DF. For examples, the *Huixi Medical Records* and *Shenji Chuyan* (Cautious Remarks on Diseases) attribute “seasonal epidemic heat toxin” to heatstroke rather than to a viral infec-tion. Likewise, *Zhouhou Beiji Fang* (Handbook of Prescriptions for Emergencies) provides the following account:

“In recent years, there has been an epidemic of eruptive sores (*ban chuang*) affecting the head, face, and body, spreading rapidly. The lesions resemble fire sores, filled with white fluid, and continuously rupture and regenerate. Without prompt treatment, severe cases may die within days. Even after recovery, the scars remain purplish-black and fade only after a year.” This description is more consistent with contagious pustular diseases or cutaneous anthrax than with DF.

Therefore, when referencing TCM descriptions of *Tianxing fever* in dengue-related research, it is crucial to conduct careful differential interpretation based on clinical manifestations and pathological characteristics. Such analytical rigor ensures both historical accuracy and scientific validity in correlating traditional disease concepts with modern clinical entities.

## 4 Molecular biology and pathogenesis of DENV

### 4.1 Molecular biology

DENV belongs to the genus *Flavivirus* within the family *Flaviviridae* (Wilder-Smith et al., 2019). Mature virions measure approximately 50 nm in diameter (≈45–55 nm), whereas immature particles are slightly larger, averaging around 60 nm (Kuhn et al., 2002; Simmonds et al., 2017). The viral genome is a ∼11 kb positive-sense single-stranded RNA, encoding a single open reading frame flanked by highly structured 5’ and 3’ untranslated regions (UTRs) that play critical roles in viral replication and translation (Pierson and Diamond, 2020). Within the 3’UTR, a double-stranded stem-loop-pseudoknot (SL-PK) structure facilitates viral host adaptation between mosquito and human hosts (Villordo et al., 2016). This region generates subgenomic flaviviral RNA (sfRNA) incouding DVxrRNA1, which inhibits the cellular XRN1 exonuclease, thereby suppressing the host’s antiviral response (Chapman et al., 2014a; Chapman et al., 2014b; Kieft et al., 2015; Villordo et al., 2015). A previous study demonstrated that the interaction between XRN1 protein and viral RNA leads to the generation of sfRNA and repressing XRN1. The resulting sfRNA, in return, represses XRN1 exonuclease activity, thereby weakening the host antiviral response and promoting viral pathogenicity (Moon et al., 2012). The 5’UTR also plays a key regulatory role: its large stem-loop A (SLA) structure functions as a promoter, coordinating viral RNA synthesis and interactions with host factors (Lodeiro et al., 2009). A small hairpin structure (sHP) located in the 3’ stem loop (3’ SL) of DENV 5’ -3’ UTRs that when DENVs carrying nucleotide changes at the loop (Mut L) or the stem (Rev3) of the sHP obtained in mammalian cells were used to infect mosquito cells, no viral replication was detected (Villordo and Gamarnik, 2013). Another study has found that the mutations in M132, MSt-1, MSt-2, and M134 (in the DEN-SL II of 3’ UTRs) increased viral RNA amplification in mosquito cells by 15–36 times, while reducing BHK and replication in human cells (Villordo et al., 2015). These suggests that 3’UTR bidirectionally influences DENV adaptation in mosquito and mammalian cells through specific sites mutations. The ORF is translated into a single polyprotein, which is post-translationally cleaved into three structural proteins—capsid (C), precursor membrane (prM) (cleaved to M during maturation), and envelope (E)—and seven nonstructural proteins (NS1, NS2A, NS2B, NS3, NS4A, NS4B, and NS5) (Nncube et al., 2018). The E and M proteins, together with a lipid bilayer, constitute the viral envelope, while the C protein provide a high-molecular-weight scaffold for genome packaging (Rey, 2013; Zhang et al., 2013; Kostyuchenko et al., 2016; Boon et al., 2023). The prM protein is emphasizing the role of prM-specific monoclonal antibodies in facilitating viral entry and linked to antibody-dependent enhancement (ADE) of DENV infection (Li et al., 2008). A study has found that the degree of neutralizing response to DENV is related to the expression level of prM and the cell line. In mosquito-derived cells (C6/36), the lower the expression of prM in DENV, the less likely it is to be neutralized when encountering prM antibodies; in human monocyte-derived cells (dendritic cells, DCs), anti-prM antibodies cannot fully neutralize the virus produced by DCs, but they can still slightly enhance infection (Dejnirattisai et al., 2010). The E protein is a key antigen in DENV vaccine development because of the most critical target of neutralizing antibodies (Dai et al., 2021; Liu et al., 2024). The nonstructural proteins are multifunctional and crucial for viral replication, immune evasion, and pathogenesis. The modulate host immune defenses primarily by interfering with interferon (IFN) signaling pathways and disrupting pattern recognition receptor (PRR)-mediated detection, resulting in enhanced viral replication and attenuated antiviral responses (Ashour et al., 2010; Green et al., 2014; Dalrymple et al., 2015; Malavige and Ogg, 2024). The NS5 and 5A proteins of *Flaviviridae* viruses for evading the innate immune response by inhibiting pattern recognition receptor (PRR) signaling pathways (TLR/MyD88, IRF7), suppressing interferon (IFN) signaling pathways (IFN-γRs, STAT1, STAT2), and impairing the function of IFN stimulated genes (ISGs) (Chen et al., 2018). NS1 is critical for immunogenicity that, evades the host immune system through a variety of mechanisms, such as complement pathway activation or inhibition and the elicitation of autoantibodies that cross-react with platelets and endothelial cells, resulting in endothelial dysfunction (Yin et al., 2013). In addition, NS1 can also trigger T cell immune responses. For example, CD4 T cell responses targets mainly C protein followed by E,NS1, NS3, NS2A/B, and NS5 proteins (Tian et al., 2019).

### 4.2 Pathogenesis

#### 4.2.1 Description of DF mechanism in TCM

Classical TCM texts do not explicitly describe a disease named “dengue fever.” However, the prevailing scholarly consensus classifies DF within the TCM framework of epidemic diseases (*yi bing*), attributing its etiology to infection by epidemic pathogenic toxins (*yi du xie qi*). The core pathogenesis is characterized by heat toxin combined with dampness (*re du jia shi*), which disturbs the nutrient and blood levels (*rao ying dong xue*) and leads depletion of qi and yin (*hao qi shang yin*) (National Health Commission of the People’s Republic of China, and National Administration of Traditional Chinese Medicine, 2024). Based on clinical manifestations such as rash, hemorrhage, and epidemic prevalence, DF is also referred to in TCM literature as “epidemic rash” (*yi zhen*) or “epidemic maculopathy” (*yi ban*) (Mucheng and Shengjie, 2012). Jiaming (1994) suggested that disease entities described in febrile-disease treatises as “summer warmth” (*shu wen*), “summer epidemic” (*shu yi*), and “epidemic rash” (*yi zhen*) may partially correspond to DF. Furthermore, TCM scholars have proposed various classifications of the disease according to seasonal occurrence and pathogenic characteristics. For example, Zhiyun (1994) divided it into three major categories: “summer-heat epidemic” (*shu re yi*), “summer-dryness epidemic” (*shu zao yi*), and “damp-heat epidemic” (*shi re yi*). Shengquan (1981) further described cases presenting with lingering summer-dampness symptoms—such as high fever, thick greasy tongue coating, and generalized myalgia—manifesting in late autumn as “latent summer-heat emerging in autumn” (*fu shu qiu fa*). In clinical TCM practice, the course of DF is typically divided into three stages—febrile, critical, and recovery—each associated with distinct pathophysiological mechanisms. During the febrile phase, the predominant pattern involves damp-heat stagnation (*shi re yu e*) and simultaneous involvement of the defensive and qi levels (*wei qi tong bing*). In the critical phase, toxin accumulation and blood stasis intertwine (*du yu jiao jie*), disrupting the nutrient-blood level (*rao ying dong xue*). During the recovery phase, residual pathogens persist (*yu xie wei jin*), leading to dual depletion of qi and yin (*qi yin liang shang*).

#### 4.2.2 Description of DF mechanism in modern medicine

DF results from the complex interplay among viral replication, host immune responses, and genetic factors. Multiple mechanisms have been proposed to explain its pathogenesis, including ADE, oxidative stress-induced cellular injury, Toll-like receptor 2 (TLR2)-mediated cytokine induction, NS1-triggered cytokine storms, and matrix metalloproteinases (MMPs)-mediated vascular leakage (Weilong et al., 2021).

Among these, ADE mechanism, first proposed by Halstead and colleagues (Halstead et al., 1970; Halstead and O’Rourke, 1977), remains-one of the most widely accepted explanations for the increased severity of dengue infection. In this process, cross-reactive or sub-neutralizing antibodies bind to heterologous DENV serotypes, facilitating viral entry into Fc receptor-bearing cells, including monocytes, macrophages, and dendritic cells. This leads to enhanced viral replication and immune activation, thereby exacerbating clinical manifestation (Wilder-Smith et al., 2019).

TLR2 and its co-receptors CD14 and TLR6 have been identified as natural receptors for DENV. Their activation induces pro-inflammatory cytokines expression and disrupts vascular integrity, contributing to endothelial dysfunction *in vitro* (Aguilar-Briseño et al., 2020). Similarly, the DENV NS1 protein can stimulate the activation of antigen-specific T cells and the production of anti-NS1 antibodies through multiple mechanisms (Wilken and Rimmelzwaan, 2020). Excessive activation of these immune cells promotes the secretion or pro-inflammatory cytokines such as TNF-α, IL-2, and IL-6, culminating in a cytokine storm that drives systemic inflammation and tissue damage in severe dengue cases (Yujie, 2005; Xiao-meng et al., 2012; Junhao, 2018).

In addition, MMPs-particularly MMP-2 and MMP-9-play key roles in increasing vascular permeability. DENV-infected dendritic cells secrete these enzymes, which downregulate the endothelial adhesion molecule PECSM-1, leading to endothelial barrier disruption, vascular leakage and F-action cytoskeletal rearrangement (Natthanej and Dorothée, 2008).

Finally, oxidative stress represents another critical pathological factor. During DENV infection, the pro-oxidative state of host cells activates transcription factors involved in inflammation, immune modulation, cellular proliferation, and apoptosis. This dysregulated oxidative response contributes to cellular damage and pathological outcomes, including excessive apoptosis or aberrant tissue remodeling (Yanping, 2010).

## 5 TCM evidence against dengue

### 5.1 Single herbs and extracts

Network-pharmacology analyses and classical TCM records collectively highlight several high-frequency herbs with potential anti-dengue properties. Literature surveys on TCM-based dengue treatment indicate that numerous Chinese herbal medicines have been traditionally applied in the management of DF (Ruting et al., 2021). In one comprehensive network pharmacology study investigating the therapeutic principles of TCM against dengue, researchers identified 10 core herbs-Cornu Bubali, Cortex Moutan, Radix Paeoniae Rubra, Rehmanniae Radix Praeparata, Lonicerae Japonicae Flos, Forsythiae Fructus, Gypsum, Scutellariae Radix, Anemarrhenae Rhizoma and Glycyrrhizae Radix et Rhizoma (Licorice)-along with 30 high-frequency herbs, which were categorized into four functional clusters:

Cluster 1: Scutellariae Radix (*Huangqin*), Glycyrrhizae Radix et Rhizoma (*Gancao*).

Cluster 2: Lonicerae Japonicae Flos (*Jinyinhua*), Forsythiae Fructus (*Lianqiao*).

Cluster 3: Coptidis Rhizoma (*Huanglian*), Cornu Bubali (*Shuiniujiao*), Gardeniae Fructus (*Zhizi*), Scrophulariaceae Radix (*Xuanshen*), Rehmanniae Radix Praeparata (*Shengdihuang*), Cortex Moutan (*Mudanpi*), Radix Paeoniae Rubra (*Chishao*), Gypsum Fibrosum (*Shigao*), Anemarrhenae Rhizoma (*Zhimu*).

Cluster 4: Radix Bupleuri (*Chaihu*), Radix Puerariae (*Gegen*), Bambusae Folium (*Zhuye*), Gypsum Fibrosum (*Shigao)*, Coicis Semen (*Yiyiren)*, Ophiopogon japonicus (*Maidong)*, Artemisia Annua (*Qinghao*), Glycyrrhizae Radix Crudus (*Shenggancao*), Talcum (*Huashi*), Pogostemon Cablin (*Huoxiang*), Isatidis Radix (*Banlangen*), Radix Rhei Et Rhizome (*Dahuang*), Isatidis Folium (*Daqingye*), Imperatae Rhizoma (*Baimaogen*), Lophatherum gracile (*Danzhuye*), Phragmitis Rhizoma (*Lugen*), Menthae Herba (*Bohe*) (Ting et al., 2024).

Among these, Scutellariae Radix (*Huangqin*), Isatidis Radix (*Banlangen*), and Isatidis Folium (*Daqingye*) are prominently documented in classical TCM texts as key treatments for *Tianxing fever*-a historical condition closely resembling dengue in its clinical features. Modern pharmacological investigations have since isolated antiviral constituents from these herbs and experimentally validated their anti-DENV activities, including inhibition of viral replication, modulation of host immune response, and reduction of inflammatory damage. Table 2 presents the classical indications for *Tianxing fever* and modern pharmacological evidence supporting their anti-dengue potential about these herbs.

**TABLE 2:** Therapeutic efficacy of a single TCM regimen against *Tianxing fever* and its extract against DENV.

Source	Extract	Mechanism of anti DENV
**Scutellariae Radix (*Huangqin*)**		
*Wan Shi Jia Chao Jishi Liangfang* (Wan’s Family Manuscript: Effective Formulas for Saving the World, 6 Volumes)	Scutellarein	The antiviral effects vary depending on concentration, manifesting as (Zandi et al., 2012a; Zandi et al., 2012b; Zandi et al., 2013): - Inhibition of viral replication; - Blockade of viral adsorption; - Direct virucidal activity.
*Jindai Neike Guoyao Chufang Ji* (Modern Internal Medicine Prescriptions with Traditional Chinese Herbs)	Baicalin	Exerts anti-DENV effects by modulating the PI3K/AKT signaling pathway, suppressing autophagy initiation and autophagosome-lysosome fusion. This reduces DENV-2-induced autophagy, thereby inhibiting viral RNA replication and NS1 protein expression (Yao et al., 2024).
**Isatidis Radix (*Banlangen*)**		
*Jindai Neike Guoyao Chufang Ji* (Modern Internal Medicine Prescriptions with Traditional Chinese Herbs)	Multiple compounds have been isolated from Isatis indigotica root, though their specific anti-DENV mechanisms require further elucidation (Gao et al., 2018)	/
**Isatidis Folium (*Daqingye*)**		
*Gujin Tushu Jicheng: Douzhen Men* (Complete Collection of Ancient and Modern Books: The Pox and Rash Section)	Water-Soluble Extract of Isatis indigotica Leaf	Exhibits inhibitory effects on DENV; Limited direct virucidal or prophylactic activity; Suppresses intracellular viral replication post-infection (Gaobo et al., 2013).
*Dongyi Baojian* (Mirror of Eastern Medicine)	Aqueous Fraction of Isatis indigotica Leaf	Demonstrates inhibitory activity against DENV (Jian, 2013).

Studies have shown that extracts of Scutellariae Radix and Isatidis Folium exhibit measurable anti-DENV activity, their underlying mechanisms partially elucidated through pharmacological and molecular analyses. In contrast, research on Isatidis Radix has thus far focused primarily on chemical component extraction, while its specific antiviral constituents and mechanisms of action against DENV remain largely undefined.

Beyond these three medicinal herbs-Scutellariae Radix, Isatidis Radix and Isatidis Folium-numerous other TCM have been historically documented as treatments for epidemic febrile diseases. A search of the “Basic Ancient Chinese Medicine Database” using the keyword “*Tianxing fever*” yielded 118 historical records. After excluding entries inconsistent with dengue-like symptoms and consolidating duplicate or cross-referenced sources, we systematically summarized herbal prescriptions and medical records describing seasonal epidemic fevers with clinical manifestations analogous to DF. Table 3 presents the summarized results including representative herbs and their classical indications.

**TABLE 3:** Single TCM used to *Tianxing fever.*

Name	Records of treating *Tianxing fever*	Source
Gypsum (*Shigao*)	This herb is known to dissipate pathogenic heat from Stomach Meridian (ST) and descend phlegm-heat from Lung Meridian (LU). Therefore, it is regarded as a cardinal remedy. Zhen Quan administered it for typhoid cases characterized by splitting headaches and flaming high fever, while Ri Hua Zi applied in the treatment of *Tianxing fever*.	*Shennong Bencao Jingshu* (Annotated Divine Farmer’s Materia Medica)
	Gypsum…Ri Hua Zi states it treats *Tianxing fever…*	*Shanghan Lun Tiaobian* (Analytical Commentary on the Treatise on Cold Damage Diseases)
Aucklandiae Radix (*Qingmuxiang*)	For *Tianxing fever* presenting with crimson-black maculae that resemb a rash, decoct 200 grams of Aucklandiae Radix (*Qingmuxiang*) in 2 liters of water until the volume is reduced to 1 liter. The resulting decoction should be administered in a single dose for therapeutic effect.	*Shennong Bencao Jingshu* (Annotated Divine Farmer’s Materia Medica)
Sophorae Flavescentis Radix (*Kushen*)	Sophorae Flavescentis Radix…soaked in glutinous rice wine during twelfth lunar month and sealed in turn can mainly treats all *Tianxing fever* with headache, thirst, body heat, even mania. Drinking one bowlful can recovery follows vomiting or sweating.	
Radix Cynanchi (*Baiwei*)	Radix Cynanchi (*Baiwei*)…For those recovered from *Tianxing fever*, or with post-illness yin deficiency/internal heat and residual heat lingering, it should be added to prescriptions according to syndrome differentiation and meridian indication.	
Radix Stephaniae Cepharanthae (*Baiyao*)	Radix Stephaniae Cepharanthae (*Baiyao*)…Cui Yuanliang’s Haishang Formula for all *Tianxing fever*: Take 10 grams of Aconitum coreanum (fine as flour), mix with a large cup of fermented rice water, take in one draught and lie supine before eating meals.	
Isatidis Radix Seed (*Lanshi*)	Isatidis Radix Seed (*Lanshi*)…Ri Hua Zi further states it treats: *Tianxing fever*, malignant boils (*ding chuang*), wandering wind (*you feng*), heat toxin (*re du*), swelling toxin (*zhong du*), wind rash (*feng zhen*); alleviates vexation/thirst, infantile malnutrition, detoxifies poisons/venomous arrows, relieves metal wounds *(jin chuang*) blood stagnation, drains pus; crucial for pediatric heat malnutrition (*re gan*) and cinnabar heat (*dan re*). Polygonum Tinctorium (*Liaolan*) is most medicinal, while Brassica oleracea (*Ganlan*) is edible for dispelling heat-induced jaundice *(re huang*).	
Golden Juice (Feces) *(Fenqing*)	Golden Juice (*Fenqing*): In twelfth lunar month, cut fresh bamboo, remove green cortex, put the feces into the bamboo and soak to extract sap. This can treats *Tianxing fever*, heat disorders *(re ji*), poisoning, malignant sores, and mushroom toxin *(xun du*). Soap pod and sugarcane in the sap can treats *Tianxing fever*.	*Zhengzhi Zhunsheng* (Patterns and Treatment Guidelines)
Natrii Sulfas (*Mangxiao*)	Natrii Sulfas (*Poxiao*): Can treats *Tianxing fever*, headache, cold-heat pathogens, abdominal distension, epidemics, jaundice, dysuria (*lin bi)*, scrofula, red eyes with nebula, reduces abscesses, drains pus, moistens hair.	*Zengding Bencao Huizuan* (Revised Compendium of Materia Medica)
Boehmeriae Rhizoma Et Radix (*Zhumagen*)	Boehmeriae Rhizoma Et Radix (*Zhumagen*): It can treats *Tianxing fever* with extreme thirst/manic behavior, threatened miscarriage with bleeding, various lin syndromes (strangury with blood). Use by apply externally to treats *Chiyou Dandu* (migratory erysipelas), carbuncles/back abscesses, traumatic wounds (staunches bleeding and scabbing), fish/chicken bone obstruction.	*Bencao Beiyao* (Essentials of Materia Medica)
	Boehmeriae Rhizoma Et Radix (*Zhumagen*): It can mainly treats Pediatric *chidan* (cinnabar heat, acute infantile rashes); Carbuncles/back abscesses; Malignant sores with sinus tracts; Poisoned arrows/snake bites; Threatened miscarriage with fetal movement; Pre/postpartum vexation; *Tianxing fever*; Manic thirst; Neutralizes mineral/metal toxins.	*Wan Shi Jia Chao Jishi Liangfang* (Wan’s Family Manuscript: Effective Formulas for Saving the World, 6 Volumes)
Musa basjoo Root (*Bajiaogen*)	Musa basjoo Root (*Bajiaogen*): this can treats *Tianxing fever*, Diabetes-like syndrome (*xiao ke*); Postpartum blood distension.	*Bencao Beiyao* (Essentials of Materia Medica) *Tuzhu Bencao Fang Hebian* (Illustrated Compendium of Materia Medica Formulas)
Trichosanthes cucumeroides (*Tuguagen*)	Trichosanthes cucumeroides (*Wanggua*): Bitter-cold. Drains heat and promotes diuresis. Treats: *Tianxing fever*; Jaundice with *xiaoke* (pound for juice intake); Frequent urination/leukorrhea…	*Bencao Beiyao* (Essentials of Materia Medica) *Shanghan Lun Tiaobian* (Analytical Commentary on the Treatise on Cold Damage Diseases)
Fossilized crab (*Shixie*)	Fossilized crab (*Shixie*): this can treats Glaucoma (*Qingmang*), *Tianxing fever*; Neutralizes all mineral toxins. Apply externally: Grind with vinegar to treats abscesses.	*Bencao Beiyao* (Essentials of Materia Medica) *Bencao Congxin* (Revised Materia Medica)
Feces with bamboo-processed licorice (*Renzhonghuang*)	Feces with bamboo-processed licorice (*Renzhonghuang*): this can treats: *Tianxing fever*; Smallpox with blood-heat causing black depressed lesions.	*Bencao Beiyao* (Essentials of Materia Medica)
Coptidis Rhizoma (*Huanglian*)	Coptidis Rhizoma (*Huanglian*): This can mainly treats Hyperactive heart fire; sudden ocular disorders…*Tianxing fever*.	*Leigong Paozhi Yaoxing Jie* (Lei Gong’s Explanations on Medicinal Properties and Processing)
Polygonati Odorati Rhizoma (*Yuzhu/Weirui*)	Polygonati Odorati Rhizoma (*Weirui*): This can mainly treats stroke-induced wind-heat; Ri Hua Zi saids it can treats alleviates mental vexation, stops cough, moistens heart-lung; Targets *Tianxing fever…*	*Shanghan Lun Tiaobian* (Analytical Commentary on the Treatise on Cold Damage Diseases)

As an empirical medical system, TCM has demonstrated notable therapeutic efficacy in the treatment of various diseases, even in the absence of clearly defined chemical compositions or molecular mechanisms underlying its antiviral and immunomodulatory effects. Clinical outcomes guided by classical prescriptions highlight TCM’s pragmatic and holistic approach, offering valuable insights for both clinical medication guidance and novel drug discovery. Future research on anti-dengue therapeutics can build upon the herbal candidates listed in Table 2, focusing on the isolation and characterization of bioactive components and on mechanistic studies employing modern pharmacological and virological methodologies.

Moreover, recent studies have identified additional natural herbs exhibiting confirmed anti-DENV activities. Table 4 presents their active constituents and mechanisms of action.

**TABLE 4:** Natural Chinese herbal medicines and pharmacological components with Anti-DENV effects.

Name	Components of Anti-DENV	Mechanism of Anti-DENV	References
Licorice (Gancao)	n-Butanol Extract of Glycyrrhiza uralensis	Targets the envelope protein of DENV-2 to inhibit viral adsorption during the replication cycle	(Lingzhu, 2022)
Artemisia annua (Qinghao)	Artesunate	Suppresses DENV-2 replication at the cellular level	(Peiyang et al., 2021)
	Artemisia annua leaf extract	Exerts potent inactivation effects against both Plasmodium parasites and dengue virus.	(Sharma et al., 2014)
	Artemisinin Combined with Artesunate	Demonstrates synergistic therapeutic effects against co-infections of dengue fever and malaria.	(Rathypriya et al., 2017)
Lonicerae Japonicae Flos (Jinyinhua)	Quercetin, Luteolin, Kaempferol, etc.	Exhibit multi-component, multi-target, and multi-pathway therapeutic characteristics in dengue treatment, primarily involving modulation of inflammatory responses and immune regulation pathways.	(Chengxin et al., 2023)
Myristicae Semen (Roudoukou)	Malabaricones C (3) and E (4)	Act as moderate-to-potent NS2B/NS3 protease inhibitors, forming hydrogen bonds with residues Gly151, Gly153, Ser135, and Asp129.	(Sivasothy et al., 2021)
Fraxini Cortex (Qinpi)	Fraxetin	Primarily inhibits DENV during the viral entry phase, suppresses viral RNA replication, reduces viral protein expression, and diminishes virion assembly.	(Feng, 2022)
Medicago polymorpha	Tetrahydroberberine (THB)	Exerts therapeutic effects against dengue fever by inhibiting secretory phospholipase A2 (sPLA2) activity.	(Hemalika et al., 2025)
Carica papaya	Papaya Leaf Extract	Synergizes with dengue virus-neutralizing plasma to inhibit the NS2B/NS3 serine protease critical for viral replication.	(Fernando et al., 2024)
Herbahypericiperforati	Hypericin	Inhibits viral replication by interfering with RNA synthesis.	(Huixing and Xueli, 2023)

### 5.2 Multi-herb formulas

The formulation design of TCM follows the classical principle of “sovereign (*jun*), minister (*chen*), assistant (*zuo*), and envoy (*shi*),” which emphasizes hierarchical coordination and synergistic interaction among multiple herbs. Such multi-herb formulations exhibit unique therapeutic advantages in the treatment of DF. Formulas composed of herbs with heat-clearing and detoxifying (*qing re jie du*) as well as blood-cooling and hemostatic (*liang xue zhi xue*) properties have been clinically observed to shorten disease duration and alleviate symptoms such as fever, rash, and hemorrhagic tendencies.

Clinical TCM texts document numerous prescriptions for treating *Tianxing fever*, many of which, including their modified variants, remain in modern clinical use for dengue management. Furthermore, pharmacological studies have begun to elucidate the anti-DENV mechanisms underlying several of these traditional formulations, including inhibition of viral replication, modulation of immune responses, and reduction of inflammatory damage. Following the screening methodology described in section 4.1, classical records of seasonal epidemic fever were systematically analyzed, and prescriptions with symptom profiles consistent with DF were retained. Table 5 presents the therapeutic formulas identified through its process.

**TABLE 5:** Prescriptions for treating *Tianxing fever* in TCM’s ancient books.

Name	Records of treating *Tianxing fever*
***Waitai Miyao* (Medical Secrets from the Royal Library)**	
*Kushen Tang* (Sophora Root Decoction)	Person who has *Tianxing fever* for cases persisting 5–6 days, should take *Kushen Tang* (Sophora Decoction).
*Yinchen Wan* (Artemisia Capillaris Pill)	For *Tianxing fever* developing jaundice after 7–8 days: Yellowing of face or body, chest oppression with panting, coarse rapid breathing, should take *Yinchen Wan* (Artemisia Capillaris Pill).
*Chi Niao Tang* (A formula composed of scallion white, fermented beans, and child urine)	People who have headache, myalgia, high fever. Disease progression: affecting hair or skin surface at first day, penetrating skin layer in second day; entering muscle tissue in third day, would avoid purgation and should take dminister *Chi Niao Tang*.
*Chaihu Tang* (Bupleurum Decoction)	If unresolved after *Chi Niao Tang*, should proceed with *Chaihu Tang* (Bupleurum Decoction).
*Guadi San* (Melon Pedicle Powder)	To ersistent *Tianxing fever* for 4–5 days, people who have unquenchable thirst, epigastric tension (palpation intolerable), insomnia, delirium. Indicates jaundice — do not wait for bloodletting. For chest constriction/insomnia, take this decoction; vomiting brings cure.
*Ganfen Tang* (Dried Feces Decoction)	Exhausted patients with saw-like dry tongue, extreme thirst, dysphagia — use Ganfen Tang (Dry Excrement Decoction), alias *Poguan Tang* (Coffin-Breaking Decoction), for extreme heat.
*Biejia Tang* (Turtle Shell Decoction)	Furthermore, for the treatment of *Tianxing fever* persisting beyond 7 days, characterized by unyielding fever, constipation or sluggish stool, fullness and stuffiness in the chest and abdomen, inability to ingest food or drink, mental confusion with delirium and incoherent speech, as well as a deep and fine pulse—among these critical manifestations, if no other treatment proves effective, can take *Biejia Tang* (Turtle Carapace Decoction).
***Zhengzhi Zhunsheng* (Patterns and Treatment Guidelines)**	
*Yi Bu Yi San Yi Jiang Fang*	If someone have *Tianxing fever*, the symptoms of all sick people would identical. Can take *Yi Bu Yi San Yi Jiang Fang*.

In TCM clinical practice, the choice of prescription is guided by the pathogenesis characteristic of each stage of the disease.

During the febrile phase, the pathogenesis is dominated by damp-heat stagnation (*shi re yu e*) and simultaneous involvement of the defensive and qi levels (*wei qi tong bing*).

In the critical phase, toxin accumulation and blood stasis intertwine (*du yu jiao jie*), disturbing the circulation of qi and blood (*qi xue yun xing*).

During the recovery phase, residual pathogens persist (*yu xie wei jin*), resulting in dual depletion of qi and yin (*qi yin liang shang*). Accordingly, different prescriptions are selected to address the distinct pathophysiological mechanisms of each stage.

Current research on TCM prescriptions for DF primarily focuses on two aspects: Clinical efficacy studies, which assess whether a prescription can alleviate symptoms, shorten disease duration, or reduce complications; and pharmacological mechanism studies, which explore the molecular and cellular pathways through which these formulas exert anti-DENV effects.

Table 6 presents a comprehensive summary of existing research on TCM prescriptions related to DF, encompassing both clinical and experimental finding.

**TABLE 6:** TCM formulas for modern treatment of DF.

Name	Treatment effect	References
**Improve symptoms**		
*Qingqi Liangying* Decoction	Demonstrates significant antipyretic effects and alleviates clinical symptoms.	(Yi et al., 2003)
*Ganghuo Qingwen* Decoction	Prevents and treats dengue fever-associated vascular endothelial dysfunction.	(Chengxin et al., 2024)
*Jiedu Huashi* Decoction	Effective for treating mild dengue fever, particularly notable for rapid fever reduction.	(Na et al., 2019)
*Haoqin Qingdan* Decoction	Modulates immune dysregulation, controls body temperature, and reduces complications.	(Shurong and Zhiyun, 2018)
*Yinqiao* Powder	Fumigation therapy for mild dengue fever: Reduces fever and alleviates headache and myalgia.	(Yongping et al., 2016)
*Xinjia Xiangru Yin* Combined with *Chaige Jieji* Decoction (Modified)	Relieves dengue-induced muscle pain.	(Xinkai et al., 2021)
*Shufeng Jiedu* Capsule	Rapid therapeutic onset, shortens disease course, and minimizes complications.	(Jianzhi et al., 2015)
*Gegen Qinlian* Decoction	Effective in treating 15 cases of dengue fever with diarrhea and fever as primary symptoms.	(Jiaxin, 2015)
Dengue Fever Decoction I	Effective against dampness-heat stagnation type dengue fever: Alleviates gastrointestinal symptoms and reduces fever.	(Jinyu et al., 2009)
*Jiedu Zhiyang* Formula	Significantly reduces rash itching and accelerates rash resolution.	(Liansheng et al., 2015)
*Tanreqing* Injection	Mitigates organ damage in early-stage patients and shortens disease duration.	(Zhongbao et al., 2021)
*Xingnaojing* Injection	Rapidly lowers body temperature.	(Mei, 2016)
Anti-DENV		
*Xia Sang Ju* Granules	Pre-administration in vitro shows potent anti-DENV-1 activity comparable to ribavirin.	(Lei et al., 2019)
*Qingxing* Granules	Exerts antipyretic and analgesic effects by suppressing inflammatory cytokines and inhibiting DENV replication in C6/36 cells, potentially via the PERK/ATF6 pathway in the unfolded protein response (UPR).	(Ying, 2023)
*Chaishi Jiedu* Granules	Inhibits DENV replication, reduces inflammation, enhances immunity, and modulates metabolic pathways.	(Zhiran et al., 2022)
*Xiyanping* Injection	Suppresses viral replication, promotes viral clearance, and boosts immune response.	(Jiachun et al., 2017)
*Xuebijing* Injection	Combats inflammation and viral activity while protecting against DENV-2 induced vascular leakage.	(Zonghui, 2022)

## 6 Conclusion

As a major global mosquito-borne infectious diseases, DF has exhibited alarming upward trends in recent years, characterized by expanding geographic distribution and increasing clinical severity. In the absence of effective antiviral therapies and amid suboptimal vaccine coverage, TCM has emerged as a promising avenue for anti-dengue research owing to its multi-target mechanisms and low risk of drug resistance. This study examined classical TCM texts to identify *Tianxing fever*—a historical disease exhibiting clinical and epidemiological parallels to dengue-and systematically compiles its therapeutic herbs, while also reviewing contemporary pharmacological studies on TCM-based anti-dengue treatment.

Findings indicate that although certain natural herbal compounds have been investigated for their antiviral constituents and mechanisms of action, the pharmacological properties of most TCM herbs remain poorly characterized. Research on multi-herb formulations has predominantly emphasized clinical efficacy observations, with limited mechanistic exploration of antiviral pathways and molecular targets. This knowledge gap restricts the broader application and scientific generalizability of TCM-based therapies. To advance the field, future research should prioritize:

1. Elucidating pharmacologically active constituents in underexplored herbs (e.g., Isatis indigotica root).
2. Deciphering synergistic mechanisms within classical multi-herb formulations.
3. Advancing translational research to bridge empirical TCM knowledge with modern virological and pharmacological validation.

By integrating traditional medical insights with modern scientific methodologies, this holistic approach holds promise for the development of novel dengue therapeutic strategies and may contribute to the broader advancement of precision medicine in infectious disease management.

## 7 Prospect

TCM texts historically relied on observational techniques (inspection, auscultation and olfaction, inquiry, and palpation) to document herbal treatments. The TCM principle of “treating different diseases with the same method” however, highlights a key limitation: similar symptoms can indicate different underlying diseases, making it difficult to accurately identify treatments for specific historical conditions like *Tianxing fever* based on textual descriptions alone. Additionally, ancient sourcing and processing methods for some herbs, particularly those involving substances like *Renzhonghuang* and *Fenqing*, may not meet modern standards of applicability or patient acceptability. Therefore, critical need exists to integrate modern research techniques with documented TCM herbs. This integration is essential to bridge historical knowledge with contemporary science, ensuring the development of safe, effective, and relevant therapies.

**FIGURE 1:**
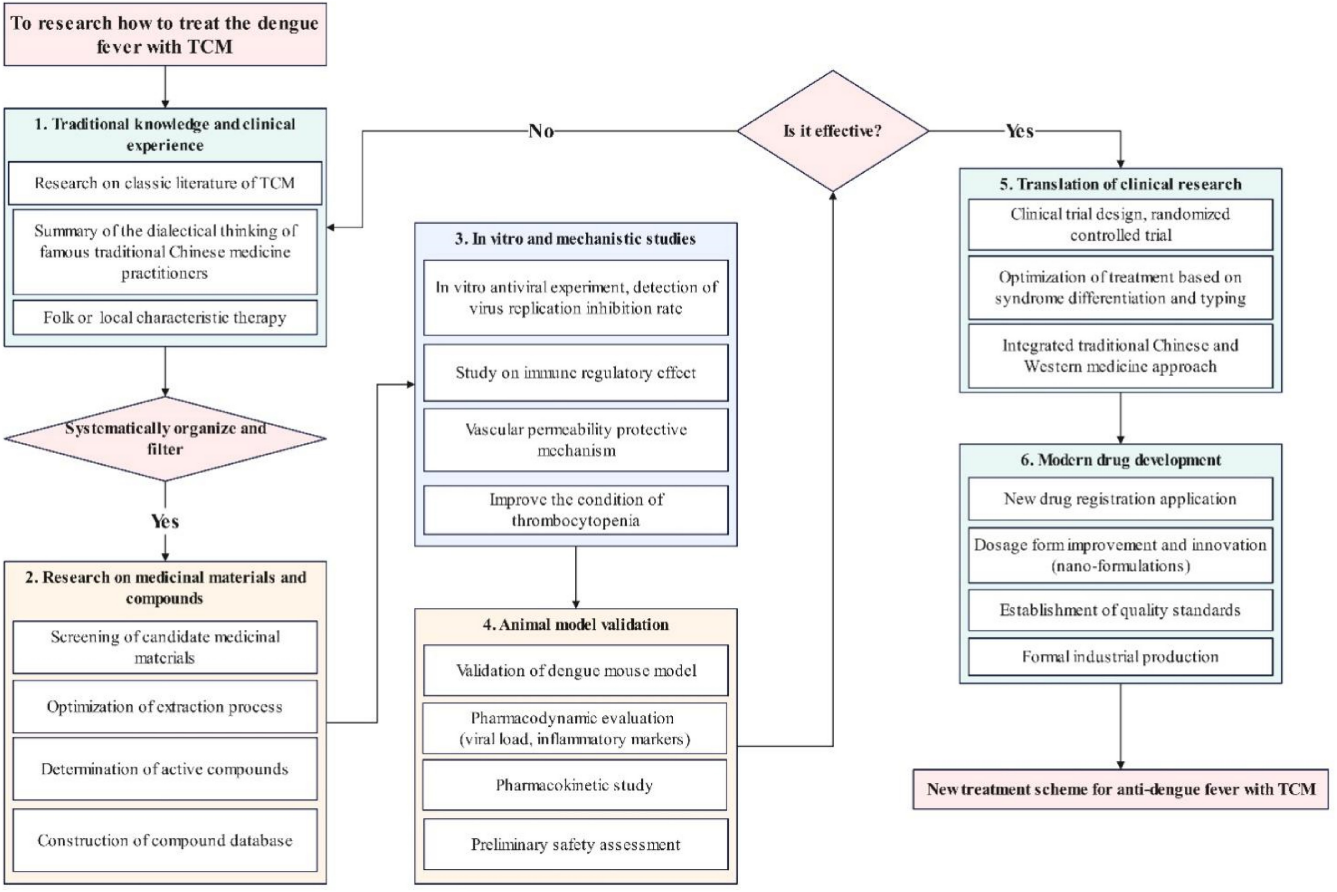
Flowchart on the research approach of TCM for dengue fever treatment.

Fortunately, research on TCM against the DENV is gradually transitioning from empirical practice to precision medicine. Beyond conventional mechanistic studies and clinical research, the exploration of TCM for DF treatment encompasses several other critical directions. These include the systematic consolidation of knowledge from experienced senior TCM practitioners, in-depth investigation of classical TCM literature, the construction of comprehensive compound databases, and modern drug research and development. To provide a clear visual overview, we have organized these interconnected research avenues into a flowchart, as shown in Figure 1. Despite persistent challenges-including compositional complexity, uncertain pharmacokinetics, and mechanistic ambiguity-TCM holds strong significant potential to deliver safer and more effective therapeutic options. This transformation is increasingly driven by the integration of modern biotechnology, innovative drug-delivery systems, and rigorous clinical validation (Lin et al., 2023).

### 7.1 Research prospects of molecule compounds in the treatment of DF

Nanotechnology offers transformative strategies to overcome the low bioavailability of many TCM compounds (Xiao, 2020). In particular, liposomes represent a promising nanocarrier platform owing to their scalability, biocompatibility, and ease of manufacturing compared with other nanocarriers. Through surface functionalization with diverse moieties, liposomes can achieve targeted delivery, controlled release, localized therapeutic action, and optimized pharmacokinetic profiles. Encapsulating plant-derived natural products in functionalized liposomes not only enhances their stability and targeting precision, while reducing systemic side effects, but also masks unpleasant odors, thereby improving patient compliance (Chengyun et al., 2024).

In addition to experimental, computational methods such as molecular docking, drug-likeness prediction, structure–activity relationship (SAR) analysis, and MMPBSA (Molecular Mechanics/Poisson–Boltzmann Surface Area) calculations can significantly accelerate drug discovery and reduce development costs. For instance, a study employing molecular modeling approaches identified isomargolonone as a potential inhibitor of DENV replication through stable binding to the NS3 protein (Choudhir et al., 2025). In another investigation, pharmacophore-based virtual screening revealed that Compound 108 exhibits high compatibility with the active site of DENV-2 RdRp and is predicted to inhibit its enzymatic activity (Roney et al., 2023). Furthermore, Qian and colleagues designed a series of compounds based on an indoline structural skeleton targeting DENV infection. Their study demonstrated that the TBS group represents a promising pharmacophore for enhancing anti-DENV activity. Specifically, compounds 13 and 15 were shown to reduce RdRp enzymatic activity and establish favorable low-energy conformations within the RdRp domain of DENV2 NS5 through hydrogen bonds and hydrophobic interactions (Qian et al., 2022).

Collectively, these findings demonstrate that minor structural modifications in natural products can confer specific binding affinity toward DENV targets. This structure–activity relationship provides a promising foundation for the discovery and development of natural-product-derived anti-DENV agents.

### 7.2 The integration of artificial intelligence, data models, and TCM practices

With the rapid development of artificial intelligence (AI) technology, its application in TCM research has evolved from initial exploration into a phase of deep integration and innovation. This convergence offers a new paradigm for elucidating the complex scientific underpinnings of TCM and establishes efficient pathways for modern drug discovery and clinical practice. Contemporary AI technologies, particularly machine learning (ML) and deep learning (DL) models, have demonstrated remarkable potential in structure prediction, spectral interpretation, and metabolic pathway modeling within the context of mass spectrometry (MS)-based TCM research (Xu et al., 2025). Furthermore, the emergence of Large Language Models (LLMs) is gradually positioning them as valuable tools for TCM studies. A recent analysis in 2025 revealed that LLMs specifically developed for TCM achieve high accuracy in both TCM practice examinations and general applications (Ren et al., 2025). In addition to computational approaches, omics technologies provide critical insights into the molecular mechanisms underlying TCM therapeutics. Transcriptomic analyses—including RNA sequencing (RNA-seq), co-expression network analysis, and fluxomics—enable the elucidation of pathway regulation. Concurrently, quantitative proteomics techniques, such as tandem mass tags (TMT), isobaric tags for relative and absolute quantitation (iTRAQ), label-free methods, cellular thermal shift assay (CETSA), and thermal proteome profiling (TPP), facilitate the identification of molecular targets and the characterization of their mechanisms of action (Xie et al., 2025).

In parallel, network pharmacology provides a powerful systems-level framework for elucidating the synergistic mechanisms of TCM formulations against DENV by integrating multidimensional component-target-pathway data (Ziqing et al., 2021; Cong, 2024; Ting et al., 2025). Furthermore, emerging multi-omics strategies-which-combine network pharmacology, metabolomics, and gut microbiota analysis-are beginning to decode the scientific basis of TCM‘s multi-target therapeutic actions (Xiumin, 2022; Shangzhi, 2023; Xuting, 2024).

Future research should prioritize bioactive herbs documented in classical TCM texts, leveraging advanced biotechnological and analytical approaches to uncover their antiviral targets and mechanistic pathways. The convergence of ancestral medical wisdom with modern scientific innovation is poised to establish novel paradigms for precision dengue therapy, bridging the gap between traditional medicine and next-generation antiviral development.
